# Adaptive divergence in resistance to herbivores in *Datura stramonium*

**DOI:** 10.7717/peerj.1411

**Published:** 2015-11-26

**Authors:** Guillermo Castillo, Pedro L. Valverde, Laura L. Cruz, Johnattan Hernández-Cumplido, Guadalupe Andraca-Gómez, Juan Fornoni, Edson Sandoval-Castellanos, Erika Olmedo-Vicente, César M. Flores-Ortiz, Juan Núñez-Farfán

**Affiliations:** 1 Department of Evolutionary Ecology, Institute for Ecology UNAM, Mexico Distrito Federal, Mexico; 2 Departamento de Biología, Universidad Autónoma Metropolitana-Iztapalapa, Mexico Distrito Federal, Mexico; 3 Laboratory of Evolutionary Entomology, Institute of Biology, University of Neuchâtel, Neuchâtel, Switzerland; 4 Facultad de Estudios Superiores Iztacala, UNAM, Estado de México, Mexico

**Keywords:** Adaptive divergence, Tropane alkaloids, Leaf trichomes, Plant defense, *P*_ST_–*F*_ST_ comparison, *Datura stramonium*, Divergent natural selection, Genetic drift and restricted gene flow, Plant resistance

## Abstract

Defensive traits exhibited by plants vary widely across populations. Heritable phenotypic differentiation is likely to be produced by genetic drift and spatially restricted gene flow between populations. However, spatially variable selection exerted by herbivores may also give rise to differences among populations. To explore to what extent these factors promote the among-population differentiation of plant resistance of 13 populations of *Datura stramonium*, we compared the degree of phenotypic differentiation (*P*_ST_) of leaf resistance traits (trichome density, atropine and scopolamine concentration) against neutral genetic differentiation (*F*_ST_) at microsatellite loci. Results showed that phenotypic differentiation in defensive traits among-population is not consistent with divergence promoted by genetic drift and restricted gene flow alone. Phenotypic differentiation in scopolamine concentration was significantly higher than *F*_ST_ across the range of trait heritability values. In contrast, genetic differentiation in trichome density was different from *F*_ST_ only when heritability was very low. On the other hand, differentiation in atropine concentration differed from the neutral expectation when heritability was less than or equal to 0.3. In addition, we did not find a significant correlation between pair-wise neutral genetic distances and distances of phenotypic resistance traits. Our findings reinforce previous evidence that divergent natural selection exerted by herbivores has promoted the among-population phenotypic differentiation of defensive traits in *D. stramonium*.

## Introduction

Most species consist of a series of populations that are often phenotypically differentiated (Rice & Jain, 1985; Thompson, 2005). Heritable phenotypic differentiation in multiple traits can be effectively produced by processes like genetic drift, mutation, founder effects or population isolation (Gomulkiewicz et al., 2007). However, phenotypic differentiation in traits that contribute to individuals’ fitness may also have a spatial structure caused by varying selective regimes exerted by biotic and/or abiotic factors (Holsinger & Weir, 2009). Furthermore, stabilizing selection may promote phenotypic similarity among populations (Merilä & Crnokrak, 2001). Elucidating to what extent these processes promote character differentiation among populations is central if we are to fully understand the prevalence of among-population variation in the wild (Lynch, 1990; Althoff & Thompson, 1999; Criscione, Blouin & Sunnucks, 2006; Kelly, 2006; Gomulkiewicz et al., 2007). Here we aimed to determine if among-population variation in traits that confer resistance to herbivores in the annual plant *Datura stramonium* is consistent with a scenario of varying selection or genetic drift and restricted gene flow.

To infer whether natural selection explains the observed differentiation among populations in putatively adaptive quantitative characters (*Q*_ST_), it is necessary to contrast this hypothesis against a null model of differentiation at adaptively neutral loci (*F*_ST_; Spitze, 1993; Martin, Chapuis & Goudet, 2008; Whitlock, 2008). The detection of a significant difference between the estimators of differentiation, *Q*_ST_ and *F*_ST_, may imply adaptive differentiation among populations. The comparison of the differentiation indices has three possible outcomes each with a unique interpretation (see Table 1 in Merilä & Crnokrak, 2001). When *Q*_ST_ and *F*_ST_ are statistically equal, this implies that the degree of differentiation in quantitative traits could be produced by drift alone. This does not necessarily imply that genetic drift produced the observed phenotypic differentiation but that the roles of selection and drift are indiscernible. When *Q*_ST_ < *F*_ST_, it means that natural selection might be favoring the same phenotype across populations. Finally, when *Q*_ST_ significantly exceeds *F*_ST_, it means that directional selection is favoring different phenotypes in different populations. When *Q*_ST_ and *F*_ST_ are equal, it is expected that both indices, estimated among pairs of populations of the same species, will be positively correlated, implying isolation by distance, restricted gene flow and genetic drift (although a partial role of selection could be involved also), or high recombination between molecular neutral marker loci and quantitative trait loci (Merilä & Crnokrak, 2001). In contrast, no correlation between both indices of differentiation among local populations may implicate a role of natural selection (see ‘Discussion’).

In order to explore the signals of non-neutral evolution in quantitative traits it is necessary to estimate *Q*_ST_ and *F*_ST_. *F*_ST_ is commonly estimated by analyzing variance in allele frequency (Wright, 1951) at molecular markers, like microsatellite loci. On the other hand, to estimate *Q*_ST_ it is necessary to know the amount of additive genetic variance of quantitative traits in many local populations (Spitze, 1993). However, accomplishing the latter objective is not feasible for a large number of populations because it requires estimating the breeding values of genotypes (families) for a suite of phenotypic characters in each local population. *P*_ST_ (degree of phenotypic differentiation index) is an analogous index to *Q*_ST_ (Leinonen et al., 2006; Leinonen et al., 2013), useful for exploring if phenotypic differentiation among populations exceeds genetic differentiation in neutral markers (Merilä & Crnokrak, 2001). The use of *P*_ST_ instead of *Q*_ST_ is justified when estimates of additive genetic variance are not available (Leinonen et al., 2006; Leinonen et al., 2013; Lehtonen et al., 2009). Estimation of additive genetic variation in traits makes it necessary to obtain the phenotypic covariance between relatives (families) with an experimental common garden and/or the use of reciprocal transplant experiments to rule out the environmental effects on phenotypes. Hence, *P*_ST_ can be used as a surrogate of *Q*_ST_.

Resistance traits exhibited by plants (i.e., traits that prevent/reduce damage by natural enemies) vary widely across populations (Núñez-Farfán, Fornoni & Valverde, 2007; Züst et al., 2012). Selection exerted by herbivores is a major force driving the evolution of plants’ resistance traits (Rausher, 2001; Anderson & Mitchell-Olds, 2011; Züst et al., 2012). Thus, among-population differentiation in resistance traits is likely to be produced by spatial variation in the local selective regimes exerted by herbivores. Such spatially variable selection can be generated by among-population variation in the abundance, species composition, feeding styles, and degree of dietary specialization of herbivores to their host plants (Falconer & Mackay, 1996; Charlesworth, Nordborg & Charlesworth, 1997; Parchman & Benkman, 2002; Arany et al., 2008; Hare, 2012). *Datura stramonium* (Solanaceae) provides an optimal system for studying among-population differentiation in resistance traits. Because of its wide distribution (Mexico, Canada, United States, and Europe), *D. stramonium* is exposed to different environmental conditions and to a wide diversity of herbivore species (Weaver & Warwick, 1984; Valverde, Fornoni & Núñez-Farfán, 2001; Cuevas-Arias, Vargas & Rodríguez, 2008). Resistance against herbivores in *D. stramonium* includes leaf trichomes (Valverde, Fornoni & Núñez-Farfán, 2001; Kariñho-Betancourt & Núñez-Farfán, 2015) and tropane alkaloids (Shonle & Bergelson, 2000), of which atropine, hyosciamine and scopolamine are the most abundant (Parr et al., 1990; Kariñho-Betancourt et al., 2015). These secondary metabolites affect the activity of the neurotransmitter acetylcholine (Roddick, 1991) with negative effects on insects and vertebrate herbivores (Hsiao & Fraenkel, 1968; Krug & Proksch, 1993; Wink, 1993; Shonle, 1999; Mithöfer & Boland, 2012). Recent studies have found ample geographic variation in leaf trichome density and atropine and scopolamine concentration in central Mexico (Castillo et al., 2013; Castillo et al., 2014). However, it is unclear if selection by herbivores or neutral processes, among other factors, can account for the observed among-population differentiation in these resistance traits.

Here, we assessed to what extent population differentiation in resistance leaf traits (trichome density, atropine and scopolamine concentrations) of *D. stramonium* is accounted by neutral processes (genetic drift and restricted gene flow) or divergent natural selection. To do so, we compared the degree of phenotypic differentiation of resistance traits by means of *P*_ST_ estimated for the whole range of values of heritability, with the neutral expectation set by allelic divergence at microsatellite loci (*F*_ST_). We expect that *P*_ST_ of each resistance character would be significantly higher than the index of population differentiation in neutral molecular markers (*F*_ST_), since previous studies have detected contrasting selection exerted by herbivores on the three characters.

## Methods

### Study system

*Datura stramonium* L. (Solanaceae) is an annual herb commonly found in roadsides, cultivated areas and disturbed environments in Mexico, the United States, Canada, and Europe (Valverde, Fornoni & Núñez-Farfán, 2001; Weaver, Dirks & Warwick, 1985; Van Kleunen, Markus & Steven, 2007). In Mexico, leaves of *D. stramonium* are consumed by a dietary specialist herbivore, the chrysomelid *Lema trilineata* (Nuñez-Farfan & Dirzo, 1994), the dietary oligophagous *Epitrix parvula* (Chrysomelidae), which also feeds from other members of the Solanaceae family (Glass, 1940), and by the dietary generalist grasshopper *Sphenarium purpurascens* (Nuñez-Farfan & Dirzo, 1994). *Datura stramonium* features leaf trichomes and tropane alkaloids (atropine and scopolamine) as resistance traits against herbivory. These traits have shown heritable basis (Shonle & Bergelson, 2000; Valverde, Fornoni & Núñez-Farfán, 2001; Kariñho-Betancourt & Núñez-Farfán, 2015), and are under selection by dietary specialist and generalist herbivores (Castillo et al., 2014).

### Fieldwork

**Figure 1:**
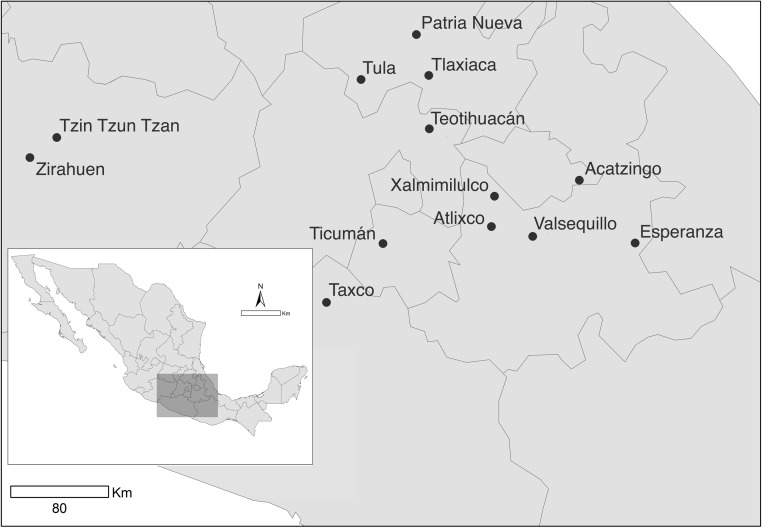
Sampled populations of *Datura stramonium* in central Mexico (see Table 1).

During August–September 2007 we sampled 13 natural populations of *D. stramonium* in central Mexico (Fig. 1). Selected populations inhabit a wide range of habitat types. The geographic location and habitat characteristics are shown in Table 1. From each population we sampled 30 randomly chosen individual plants.

**Table 1:** Vegetation type, latitude, longitude, altitude and population means of leaf trichome density, and atropine and scopolamine concentrations of 13 populations of *Datura stramonium* in central Mexico.

	Vegetation type	Latitude	Longitude	Altitude (m a.s.l.)	Trichome density (2.5 × mm^2^)	Atropine (mg/g)	Scopolamine (mg/g)
1. Acatzingo	DS	−97.78	19.32	2,160	8.99	0.295	0.159
2. Atlixco	DS	−98.42	18.98	1,840	9.04	0.691	0.577
3. Esperanza	DS	−97.37	18.85	2,278	9.57	0.535	0.542
4. Patria Nueva	DS	−98.96	20.38	2,040	12.62	0.317	0.367
5. Taxco	TDF	−99.66	18.5	1,582	9.02	0.957	0.266
6. Teotihuacán	DS	−98.86	19.68	2,294	8.73	0.437	0.353
7. Ticumán	TDF	−99.2	18.86	1,210	6.6	0.938	1.889
8. Tlaxiaca	DS	−98.86	20.08	2,340	9.36	0.288	0.458
9. Tula	DS	−99.35	20.05	2,020	6.06	3.129	2.804
10. Tzin Tzun Tzan	POF	−101.58	19.63	2,050	4.29	0.994	2.995
11. Valsequillo	DS	−98.11	18.91	2,209	6.09	1.767	0.044
12. Xalmimilulco	POF	−98.38	19.2	1,200	4.66	2.688	2.513
13. Zirahuén	POF	−101.91	19.43	2,174	4.91	0.618	1.968

**Notes.**

DS desert shrub POF Pine–Oak forest TDF tropical deciduous forest

### Resistance traits quantification

Following Valverde, Fornoni & Núñez-Farfán (2001), we estimated leaf trichome density as the total number of trichomes in an observation field of 2.5 mm^2^ located in the central basal region of the adaxial side of the leaf, using a stereoscopic microscope. Then we averaged the trichome density per plant from a random sample of 20 fully expanded leaves. We also quantified the concentration of atropine and scopolamine (two major alkaloids in *D. stramonium*) from a sample of 20 leaves per plant by means of High Precision Liquid Chromatography (HPLC). Details of the extraction method and HPLC conditions can be found elsewhere (see Castillo et al., 2013).

### Data analysis

We estimated the neutral genetic differentiation among populations of *D. stramonium* using *F*_ST_ values obtained from five nuclear microsatellite markers designed specifically for *D. stramonium* as reported by Andraca-Gómez (2009). *F*_ST_ values were calculated using FSTAT 2.9.3.1 (Goudet, 2001) employing approximately 30 individuals per population. In addition, we assessed the statistical power of our five microsatellites by means of Wright–Fisher simulations as implemented in the program PowSim (Ryman & Palm, 2006). The program requires a divergence time and effective populations sizes so we tested a number of feasible combinations.

#### Phenotypic divergence in resistance traits

We used the degree of among-population phenotypic divergence (*P*_ST_) to explore if restricted gene flow and genetic drift (*F*_ST_) alone can account for this differentiation or if there is a signal of differentiation promoted by divergent selection on resistance traits (Leinonen et al., 2006; Pujol et al., 2008). We estimated *P*_ST_ as

\begin{eqnarray*} {P}_{\mathrm{ST}}=\frac{{\sigma }_{\mathrm{GB}}^{2}}{{\sigma }_{\mathrm{GB}}^{2}+2({h}^{2}\bullet {\sigma }_{\mathrm{GW}}^{2})}, \end{eqnarray*}

where ${\sigma }_{\mathrm{GB}}^{2}$ is the variance among populations, ${\sigma }_{\mathrm{GW}}^{2}$ is the variance within population, and *h*^2^ is the trait heritability (Leinonen et al., 2006). Since this is not feasible for a large number of populations we used an approximation by *P*_ST_.

In order to obtain *P*_ST_ values for resistance traits, we simulated the whole range of heritabilities (0 ≤ *h*^2^ ≤ 1). To estimate *P*_ST_ values we fitted a linear model for each resistance trait, under the assumption that the distribution of resistance traits was normally distributed. The *population* term was considered as a random effect. To test the hypothesis that *P*_ST_ is higher than *F*_ST_, a Monte Carlo test was carried out, approaching a sample of 10,000 deviates from both *P*_ST_ and *F*_ST_ by means of their estimated error. *P*_ST_ error was estimated from the likelihood errors of its components (variances among- and within-populations), while *F*_ST_ error was obtained by bootstrapping (Goudet, 2001). The 10,000 random deviates of *F*_ST_ and *P*_ST_ were compared and the *p*-value was obtained as the proportion of comparisons in which the *F*_ST_ was equal or higher than the *P*_ST_ (null hypothesis).

We further evaluated the pair-wise Pearson’s correlation between *F*_ST_ and *P*_ST_ for all populations. Neutral marker variation can be used as a neutral expectation against which the phenotypic divergence of traits can be compared (Gomulkiewicz et al., 2007). If resistance phenotypic differentiation between populations (*P*_ST_) is the result of neutral processes rather than selection, differentiation among populations in these traits should correlate positively with differentiation in selectively neutral markers (*F*_ST_) (Merilä & Crnokrak, 2001; Gomulkiewicz et al., 2007; Lehtonen et al., 2009; Leinonen et al., 2013). We evaluated the pair-wise correlation between the *F*_ST_ and *P*_ST_ for different scenarios of heritability (*h*^2^ = 0.1, 0.25, 0.5, 0.75 and 1.0). Statistical analyses were performed using JMP^®^ version 9.0.0 (SAS Institute, Cary, NC, 1989–2007).

## Results

### Among-population variation in resistance traits

**Figure 2:**
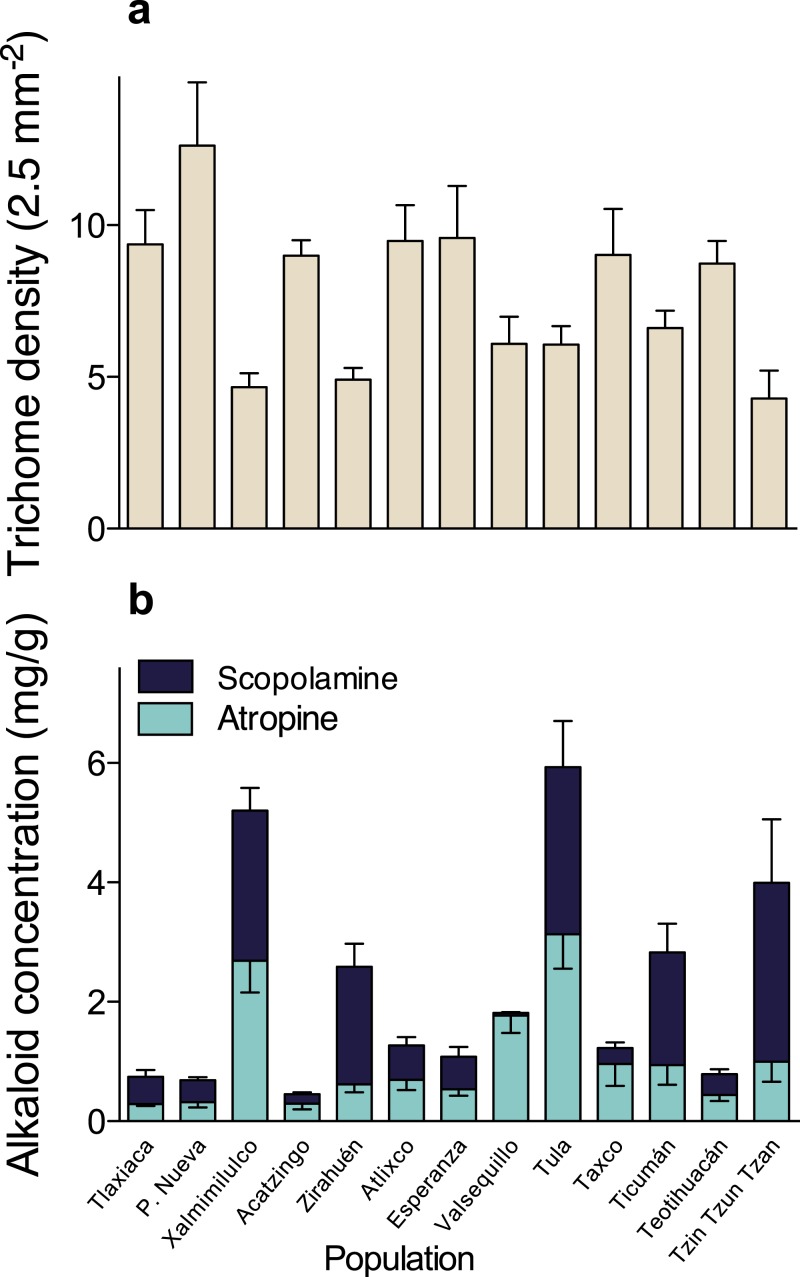
Among-populations variation in leaf trichome density (A), and atropine and scopolamine concentration (B) in 13 populations of *Datura stramonium* in central Mexico. Bars represent average value +1 SE.

A multivariate analysis of variance (MANOVA) detected significant multivariate differences in the studied resistance traits of 13 populations of *D. stramonium* (Wilks’ *λ* = 0.091, *F*_36,331.64_ = 11.51, *P* < 0.0001). After the subsequent univariate ANOVAs were applied, we found significant differences in trichome density (*F*_12,126_ = 5.10, *P* < 0.0001), atropine (*F*_12,126_ = 7.85, *P* < 0.0001), and scopolamine concentration (*F*_12,126_ = 23.33, *P* < 0.0001). Mean leaf trichome density and mean atropine and scopolamine concentration per population are shown in Fig. 2 and Table 1.

### Genetic differentiation between populations of *D. stramonium*

Genetic differentiation as estimated by differences in allele frequency at microsatellite loci was moderate. *F*_ST_ was 0.228 (S.E. = 0.039), which is well above the minimum detectable value (*F*_ST_ = 0.01) that our sample and markers allowed with a statistical power of 0.94.

### Phenotypic divergence in resistance traits

**Figure 3:**
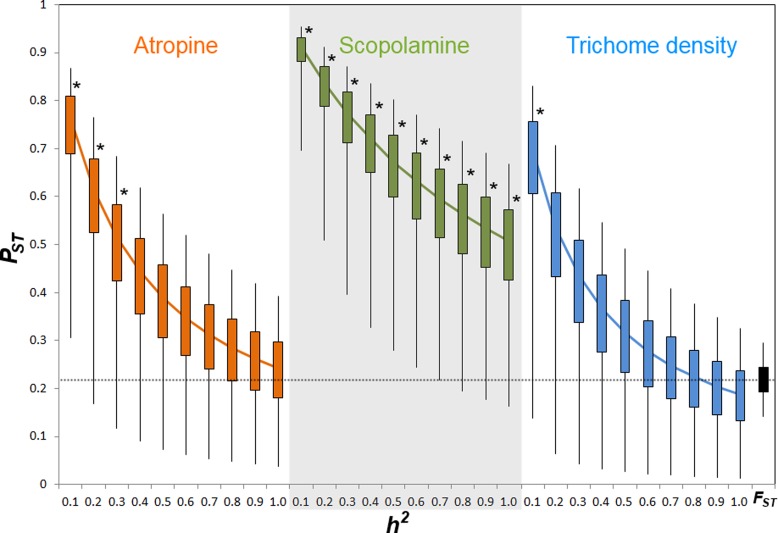
*P*_ST_ values of putative defensive traits of *Datura stramonium* as a function of their genetic variance (*h*^2^) among populations. Confidence intervals of 50% and 95% are indicated by bars and lines, respectively. * Represents overall *P*_ST_ values that differ significantly from *F*_ST_ (the black bar at the right end) after a Monte Carlo test (10,000 deviates from both *P*_ST_ and *F*_ST_; see ‘Methods’).

Comparison of phenotypic (*P*_ST_) and neutral genetic marker divergence (*F*_ST_) showed that *P*_ST_ values for scopolamine concentration were significantly higher than the *F*_ST_ in all values of *h*^2^ (Fig. 3). However, *P*_ST_ for atropine concentration was significantly higher than *F*_ST_ when 0 ≥ *h*^2^ ≤ 0.3 (Fig. 2), whereas *P*_ST_ of leaf trichome density significantly exceeded *F*_ST_ only when *h*^2^ ≤ 0.1 (Fig. 2).

### Pair-wise correlation between *F*_ST_ and *P*_ST_

We found no significant correlations between pair-wise *F*_ST_ and *P*_ST_ values among populations for any of the three resistance characters (Table 2). Most correlation values were small (i.e.,−0.135 ≤ *r* ≤ 0.034).

**Table 2:** Correlation (*r*) between pair-wise *P*_ST_ of three resistance traits and pair-wise *F*_ST_ for all populations of *Datura stramonium*, under different scenarios of heritability (*h*^2^ = 0.1, 0.25, 0.5, 0.75 and 1.0).

Resistance trait			*r*		
	*h*^2^ = 0.1	*h*^2^ = 0.25	*h*^2^ = 0.5	*h*^2^ = 0.75	*h*^2^ = 1.0
Atropine	−0.0644	−0.0671	−0.0655	−0.0642	−0.0637
Scopolamine	0.0264	0.0344	0.0348	0.0316	0.0278
Trichome density	−0.135	−0.1218	−0.1053	−0.0939	−0.0855

## Discussion

Results showed that phenotypic differentiation in resistance traits among population of *D. stramonium* is not consistent with divergence promoted by genetic drift and restricted gene flow alone (Pujol et al., 2008; Lehtonen et al., 2009). Phenotypic differentiation in scopolamine concentration was significantly higher than *F*_ST_ across the range of *h*^2^. In contrast, genetic differentiation in trichome density was different from *F*_ST_ only when heritability was very low, and most phenotypic variation could be related to major environmental factors, like annual mean precipitation and temperature. Likewise, differentiation in atropine concentration seems to differ from the neutral expectation only at low values of *h*^2^. Furthermore, we did not find a correlation between pair-wise neutral genetic distances and phenotypic distances of any of the three resistance traits. Taken together, results suggest that natural selection could be involved in phenotypic divergence on resistance traits among populations of *D. stramonium*.

Results indicate that populations of *D. stramonium* are differentiated in both phenotypic and neutral molecular markers. We found a moderate amount of differentiation among populations at microsatellite loci (*F*_ST_ = 0.228). Using this *F*_ST_ value, the indirect estimate of gene flow (*Nm*) is 0.846, suggesting restricted gene flow among populations of *D. stramonium*, and not sufficient to prevent differentiation by genetic drift (Hedrick, 2000). This contrasts with differentiation at neutral loci reported for other organisms where *F*_ST_ is generally lower than 0.228 (but see Merilä & Crnokrak, 2001). *P*_ST_ index values statistically not different from this value of *F*_ST_ imply that quantitative phenotypic characters follow a pattern of drift-induced divergence (Leinonen et al., 2006). Here, we found that the *P*_ST_ index of scopolamine was significantly higher than *F*_ST_ for all values of heritability considered (cf. Fig. 3). This result strongly suggests that phenotypic differentiation among populations in scopolamine concentration is congruent with a scenario of divergent selection exerted by herbivores among populations. However, *P*_ST_ of atropine and leaf trichome density was higher than *F*_ST_ only when heritability was ≤0.3 and ≤0.1, respectively. This implies that the proportion of genetic variance among populations from total genetic variance is high for these characters (Lehtonen et al., 2009; Leinonen et al., 2013). When genetic variance within populations is low, as implied by low values of heritability, there is a high opportunity to detect a significant *P*_ST_ given that the among-population genetic variance component has a relevant weight in the total phenotypic variance. Inversely, when heritability is high, the within-population genetic component accounts for a high fraction of total genetic variance rendering *P*_ST_ very small. These considerations may explain why *P*_ST_ of trichome density and atropine are different from *F*_ST_ only at very low heritability.

Although *P*_ST_ is used as an analog of *Q*_ST_ (genetic differentiation in quantitative characters) when it is not possible to obtain the amount of additive genetic variation (variance among families, within populations) (Merilä & Crnokrak, 2001), conclusions derived from these estimations must be interpreted with caution since this index can be biased by all environmental variation due to abiotic conditions among localities as well as environmental deviations within populations, and non-additive genetic variation (v.gr., epistatic interactions, dominance, linkage disequilibrium), among others (Pujol et al., 2008). Thus is relevant to ask whether *P*_ST_ index obtained for the resistance traits in *D. stramonium* posses genetic variance. *Datura stramonium* displays a great variation among populations in trichome density and tropane alkaloids’ concentration in central Mexico (Castillo et al., 2013). Phenotypic variation in alkaloid concentration, like other quantitative traits, is governed by environmental physical factors and genetic variation (Castillo et al., 2013). Previous studies in this species have detected narrow-sense *h*^2^ of general resistance to herbivores of 0.49 and 0.41 in two natural populations of *D. stramonium* (Fornoni, Valverde & Núñez-Farfán, 2003; note that general resistance may include physical and chemical defenses). In addition, broad-sense *h*^2^ of general resistance and trichome density has been estimated in 0.25 and 0.64, respectively (Kariñho-Betancourt & Núñez-Farfán, 2015). Also, genetic variance in trichome density among-populations (Valverde, Fornoni & Núñez-Farfán, 2001) and general resistance (Valverde, Fornoni & Núñez-Farfán, 2003; Carmona & Fornoni, 2013) has been detected in *D. stramonium*. Finally, genetic variance in alkaloid concentration (hyosciamine and scopolamine, and their ratio) has been detected previously by Shonle & Bergelson (2000). Thus, there is ample evidence of genetic basis of phenotypic variation in resistance of *D. stramonium* to support our estimation of *P*_ST_ values.

Because a *P*_ST_ index higher than *F*_ST_ means that divergent selection might be involved in population differentiation of resistance traits, at least for scopolamine, it is relevant to ask to what extent natural selection by herbivores is responsible for population differentiation in this character. In *D. stramonium*, several lines of evidence strongly suggest that differentiation in resistance is accounted by for herbivores. Differential and contrasting selection gradients on resistance to herbivores were detected between two populations of this species in a reciprocal transplant experiment (Fornoni, Valverde & Núñez-Farfán, 2004). Likewise, Shonle & Bergelson (2000) detected stabilizing selection on hyosciamine and directional selection to reduce scopolamine concentration in *D. stramonium*. In a recent study of eight populations of *D. stramonium*, Castillo et al. (2014) found that atropine is selected against by the dietary specialist herbivores *Epitrix parvula* (in one population) and *Lema daturaphila* (in two populations). In contrast, scopolamine was positively selected in one population where the specialist *Lema daturaphila* was the main herbivore, whereas trichome density was positively selected in two populations (one with *L. daturaphila* and one with the generalist grasshopper *Sphenarium puprurascens*), and negatively selected in one population with the *E. parvula* (Castillo et al., 2014). Thus, although genetic drift and restricted gene flow could produce phenotypic variation in plant resistance among populations, the available evidence of spatially variable selection on resistance traits in *D. stramonium* and data presented here suggests that population differentiation can be potentially adaptive.

**Figure 4:**
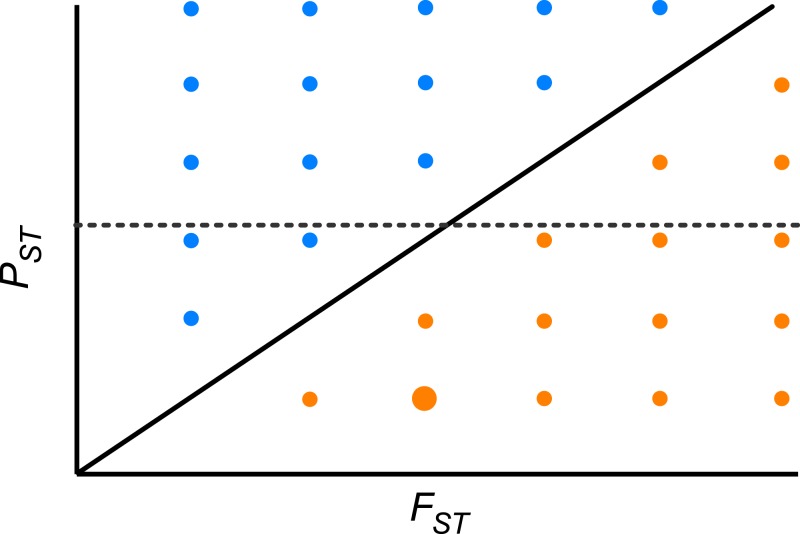
Theoretical relationship between pair-wise *P*_ST_ and pair-wise *F*_ST_ across populations of a species. The solid diagonal line indicates a perfect and positive correlation between both indices (*r* = 1, *P*_ST_ = *F*_ST_). Above the diagonal, blue points are pairs of populations where *P*_ST_ > *F*_ST_. Below the diagonal, orange points are pairs of populations where *P*_ST_ < *F*_ST_. Dotted line indicates one possible scenario where both indices are uncorrelated. At moderate values of *F*_ST_ stabilizing selection might be promoting low phenotypic differentiation between a given pair of populations (big orange point).

Furthermore, we did not detect any significant correlation between the pair-wise *P*_ST_ and *F*_ST_ among population across the whole range of heritability, suggesting that differentiation at quantitative traits and neutral molecular loci is decoupled. Theoretically, if the pace of differentiation is dictated by genetic drift only, it is expected that differentiation indices will be perfectly and positively correlated (*r* = 1, *P*_ST_ = *F*_ST_; Fig. 4). If the correlation is positive but lower than 1, then genetic drift has a role but does not explain all differentiation in quantitative traits. In the region above the diagonal in Fig. 4, where *P*_ST_ > *F*_ST_, any positive pair-wise correlation across populations, depicts a scenario where differentiation in quantitative traits exceeds the neutral expectation and suggests divergent selection (Fig. 4). On the other hand, in the region below the diagonal, where *P*_ST_ < *F*_ST_, any positive pair-wise correlation across populations, portrays a scenario where differentiation at neutral molecular loci surpasses that of quantitative characters suggesting a strong effect of genetic drift; however at moderate values of *F*_ST_ stabilizing selection might be favoring the same phenotype across populations (Fig. 4). When *P*_ST_ > *F*_ST_ and are uncorrelated (dotted line in Fig. 4) it shows another interesting scenario, as found here. This implies that genetic drift and restricted gene flow alone cannot explain (Pujol et al., 2008) the pattern of differentiation among populations in resistance traits in *D. stramonium*. Under this scenario there is opportunity for divergence driven by selection in resistance traits. Our results suggest that the higher *P*_ST_ than *F*_ST_ for scopolamine, together with spatial variation in resistance traits and the existence of a selection mosaic detected previously by Castillo et al. (2014) are consistent with outcomes predicted by the geographic mosaic of coevolution (Thompson, 2005).

## Supplemental Information

10.7717/peerj.1411/supp-1 Supplemental Information 1 Defensive characters in *Datura stramonium* Data of trichome density, atropine and scopolamine concentration in plants of *Datura stramonium* from 13 populations in Central Mexico.

## Additional Information and Declarations

### Data Availability

The following information was supplied regarding data availability: The research in this article did not generate any raw data.
